# Supplementary material for: Pyruvate Dehydrogenase Kinase 1 inhibition mediated oxidative phosphorylation enhancement in cartilage promotes osteoarthritis progression
Source: BMC Musculoskelet Disord. 2023 Jul 20;24:597. doi: 10.1186/s12891-023-06585-6 (PMC10357736; doi:10.1186/s12891-023-06585-6)
Supplement: Supplementary file 1 — Additional file 1. [file 12891_2023_6585_MOESM1_ESM.zip › MF.Gsea.1653627316242/GOMF_GLUCURONOSYLTRANSFERASE_ACTIVITY.html]

Details for gene set GOMF\_GLUCURONOSYLTRANSFERASE\_ACTIVITY[GSEA]

|  || Dataset | 55457\_collapsed\_to\_symbols.cls.cls #OA\_versus\_normal.cls.cls #OA\_versus\_normal\_repos |
| Phenotype | cls.cls#OA\_versus\_normal\_repos |
| Upregulated in class | OA |
| GeneSet | GOMF\_GLUCURONOSYLTRANSFERASE\_ACTIVITY |
| Enrichment Score (ES) | 0.42087302 |
| Normalized Enrichment Score (NES) | 1.3522993 |
| Nominal p-value | 0.1056338 |
| FDR q-value | 0.36239645 |
| FWER p-Value | 1.0 |
Table: GSEA Results Summary

  

Fig 1: Enrichment plot: GOMF\_GLUCURONOSYLTRANSFERASE\_ACTIVITY      
 Profile of the Running ES Score & Positions of GeneSet Members on the Rank Ordered List

  

| SYMBOL | TITLE | RANK IN GENE LIST | RANK METRIC SCORE | RUNNING ES | CORE ENRICHMENT || 1 | EXT2 | exostosin glycosyltransferase 2 [Source:HGNC Symbol;Acc:HGNC:3513] | 170 | 0.838 | 0.1228 | Yes |
| 2 | B4GAT1 | "beta-1,4-glucuronyltransferase 1 [Source:HGNC Symbol;Acc:HGNC:15685]" | 282 | 0.764 | 0.2381 | Yes |
| 3 | UGT2B4 | UDP glucuronosyltransferase family 2 member B4 [Source:HGNC Symbol;Acc:HGNC:12553] | 566 | 0.634 | 0.3190 | Yes |
| 4 | UGT2B17 | UDP glucuronosyltransferase family 2 member B17 [Source:HGNC Symbol;Acc:HGNC:12547] | 2050 | 0.356 | 0.2617 | Yes |
| 5 | CHPF2 | chondroitin polymerizing factor 2 [Source:HGNC Symbol;Acc:HGNC:29270] | 2281 | 0.329 | 0.2972 | Yes |
| 6 | CSGALNACT1 | chondroitin sulfate N-acetylgalactosaminyltransferase 1 [Source:HGNC Symbol;Acc:HGNC:24290] | 2507 | 0.302 | 0.3288 | Yes |
| 7 | UGT2A3 | UDP glucuronosyltransferase family 2 member A3 [Source:HGNC Symbol;Acc:HGNC:28528] | 2854 | 0.265 | 0.3448 | Yes |
| 8 | B3GAT3 | "beta-1,3-glucuronyltransferase 3 [Source:HGNC Symbol;Acc:HGNC:923]" | 2987 | 0.251 | 0.3753 | Yes |
| 9 | UGT1A8 | UDP glucuronosyltransferase family 1 member A8 [Source:HGNC Symbol;Acc:HGNC:12540] | 3301 | 0.221 | 0.3868 | Yes |
| 10 | EXT1 | exostosin glycosyltransferase 1 [Source:HGNC Symbol;Acc:HGNC:3512] | 3321 | 0.219 | 0.4209 | Yes |
| 11 | B3GAT1 | "beta-1,3-glucuronyltransferase 1 [Source:HGNC Symbol;Acc:HGNC:921]" | 4715 | 0.095 | 0.3281 | No |
| 12 | UGT1A10 | UDP glucuronosyltransferase family 1 member A10 [Source:HGNC Symbol;Acc:HGNC:12531] | 4850 | 0.083 | 0.3312 | No |
| 13 | EXTL1 | exostosin like glycosyltransferase 1 [Source:HGNC Symbol;Acc:HGNC:3515] | 6193 | -0.020 | 0.2303 | No |
| 14 | LARGE1 | LARGE xylosyl- and glucuronyltransferase 1 [Source:HGNC Symbol;Acc:HGNC:6511] | 7164 | -0.093 | 0.1700 | No |
| 15 | UGT2B11 | UDP glucuronosyltransferase family 2 member B11 [Source:HGNC Symbol;Acc:HGNC:12545] | 7270 | -0.100 | 0.1781 | No |
| 16 | CHPF | chondroitin polymerizing factor [Source:HGNC Symbol;Acc:HGNC:24291] | 7960 | -0.152 | 0.1492 | No |
| 17 | UGT2A1 | UDP glucuronosyltransferase family 2 member A1 complex locus [Source:HGNC Symbol;Acc:HGNC:12542] | 9451 | -0.269 | 0.0772 | No |
| 18 | CHSY1 | chondroitin sulfate synthase 1 [Source:HGNC Symbol;Acc:HGNC:17198] | 11628 | -0.521 | -0.0071 | No |
| 19 | UGT2B15 | UDP glucuronosyltransferase family 2 member B15 [Source:HGNC Symbol;Acc:HGNC:12546] | 12225 | -0.652 | 0.0524 | No |
Table: GSEA details [plain text format]

  

Fig 2: GOMF\_GLUCURONOSYLTRANSFERASE\_ACTIVITY      
 Blue-Pink O' Gram in the Space of the Analyzed GeneSet

  

Fig 3: GOMF\_GLUCURONOSYLTRANSFERASE\_ACTIVITY: Random ES distribution      
 Gene set null distribution of ES for **GOMF\_GLUCURONOSYLTRANSFERASE\_ACTIVITY**
